# Backpropagating Action Potentials Enable Detection of Extrasynaptic Glutamate by NMDA Receptors

**DOI:** 10.1016/j.celrep.2012.03.007

**Published:** 2012-05-31

**Authors:** Yu-Wei Wu, Sergei Grebenyuk, Thomas J. McHugh, Dmitri A. Rusakov, Alexey Semyanov

**Affiliations:** 1 RIKEN Brain Science Institute, Wako, Saitama 351-0198, Japan; 2 UCL Institute of Neurology, University College London, London WC1N 3BG, UK; 3 University of Nizhny Novgorod, Nizhny Novgorod 603950, Russia

## Abstract

Synaptic NMDA receptors (NMDARs) are crucial for neural coding and plasticity. However, little is known about the adaptive function of extrasynaptic NMDARs occurring mainly on dendritic shafts. Here, we find that in CA1 pyramidal neurons, backpropagating action potentials (bAPs) recruit shaft NMDARs exposed to ambient glutamate. In contrast, spine NMDARs are “protected,” under baseline conditions, from such glutamate influences by perisynaptic transporters: we detect bAP-evoked Ca^2+^ entry through these receptors upon local synaptic or photolytic glutamate release. During theta-burst firing, NMDAR-dependent Ca^2+^ entry either downregulates or upregulates an *h*-channel conductance (*G*_h_) of the cell depending on whether synaptic glutamate release is intact or blocked. Thus, the balance between activation of synaptic and extrasynaptic NMDARs can determine the sign of *G*_h_ plasticity. *G*_h_ plasticity in turn regulates dendritic input probed by local glutamate uncaging. These results uncover a metaplasticity mechanism potentially important for neural coding and memory formation.

## Graphical abstract

**Graphical Abstract:**

**Figure:**
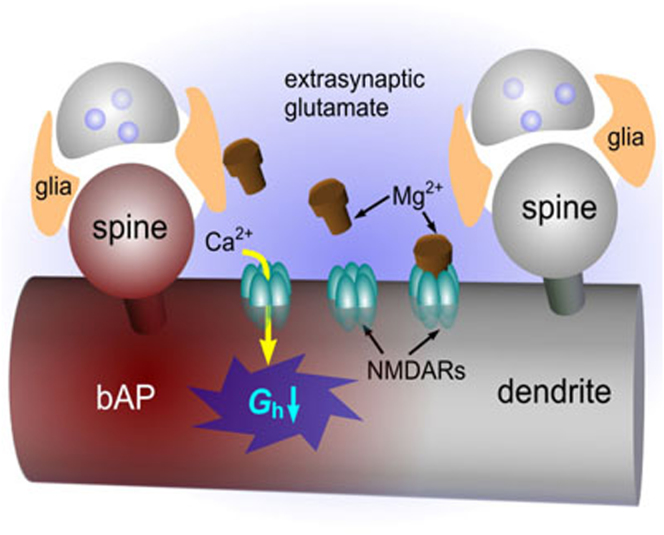

**Highlights:**

► Dendritic shaft NMDA receptors are bound to extrasynaptic glutamate ► Both dendritic shaft and spine NMDA receptors detect synaptic glutamate spillover ► Backpropagating APs help to detect both spillover and ambient glutamate ► Dendritic shaft NMDA receptors induce downregulation of *h*-channel conductance (*G*_h_)

## Teaser

Activity-dependent synaptic plasticity holds the key to information storage in the brain. Although synaptic NMDA receptors (NMDARs) have long been implicated in the underlying mechanisms, little is known about the role of extrasynaptic NMDARs. In hippocampal pyramidal neurons, backpropagating action potentials recruit extrasynaptic NMDARs that have been exposed to ambient glutamate, thus boosting local Ca^2+^ entry. Semyanov and colleagues show that physiological stimulation engaging this mechanism induces an as-yet-undiscovered form of neuronal plasticity that affects synaptic input processing by the neuron.

## Introduction

In many neurons, action potentials (APs) propagate not only into the axon but also “backward” into the dendritic processes (backpropagating APs, bAPs), where they contribute to synaptic plasticity (Magee and Johnston, 1997; Markram et al., 1997; Stuart and Häusser, 2001) and homeostatic changes in dendritic excitability (Campanac et al., 2008; Losonczy et al., 2008). In dendrites, bAPs trigger Ca^2+^ entry mainly by activating voltage-dependent Ca^2+^ channels (VDCCs) (Sabatini and Svoboda, 2000). It has also been shown that activation of synaptic NMDA receptors (NMDARs) by glutamate can be enhanced by bAPs that facilitate the removal of the NMDAR voltage-dependent Mg^2+^ block (Nevian and Sakmann, 2004; Schiller et al., 1998; Yuste and Denk, 1995).

In some physiological circumstances, glutamate molecules can escape (“spill over”) the synaptic cleft and activate NMDARs that are “shared” among neighboring synapses (Arnth-Jensen et al., 2002; Lozovaya et al., 2004a; Scimemi et al., 2004). In addition, glutamate release from astrocytes has been implicated in the activation of neuronal dendritic NMDARs (Parri et al., 2001; Shigetomi et al., 2008), suggesting a mechanism for synchronous excitation of multiple cells (Angulo et al., 2004; Carmignoto and Fellin, 2006; Fellin et al., 2004).

Nonetheless, whether and how dendritic NMDARs bound to ambient extracellular glutamate could be activated by bAPs is poorly understood. Experimental removal of the voltage-dependent Mg^2+^ block of NMDARs uncovers a tonic NMDAR-mediated current in hippocampal neurons (Cavelier and Attwell, 2005; Le Meur et al., 2007; Sah et al., 1989) even though the estimated level of ambient glutamate in quiescent hippocampal tissue is very low (Herman and Jahr, 2007). Here, we asked whether bAPs can enable dendritic NMDARs in CA1 pyramidal cells to detect and respond to activity-dependent changes in the extracellular glutamate concentration.

## Results

### NMDARs Contribute to bAP-Evoked Ca^2+^ Entry in Dendritic Shafts but Not in Spines

**Figure 1:**
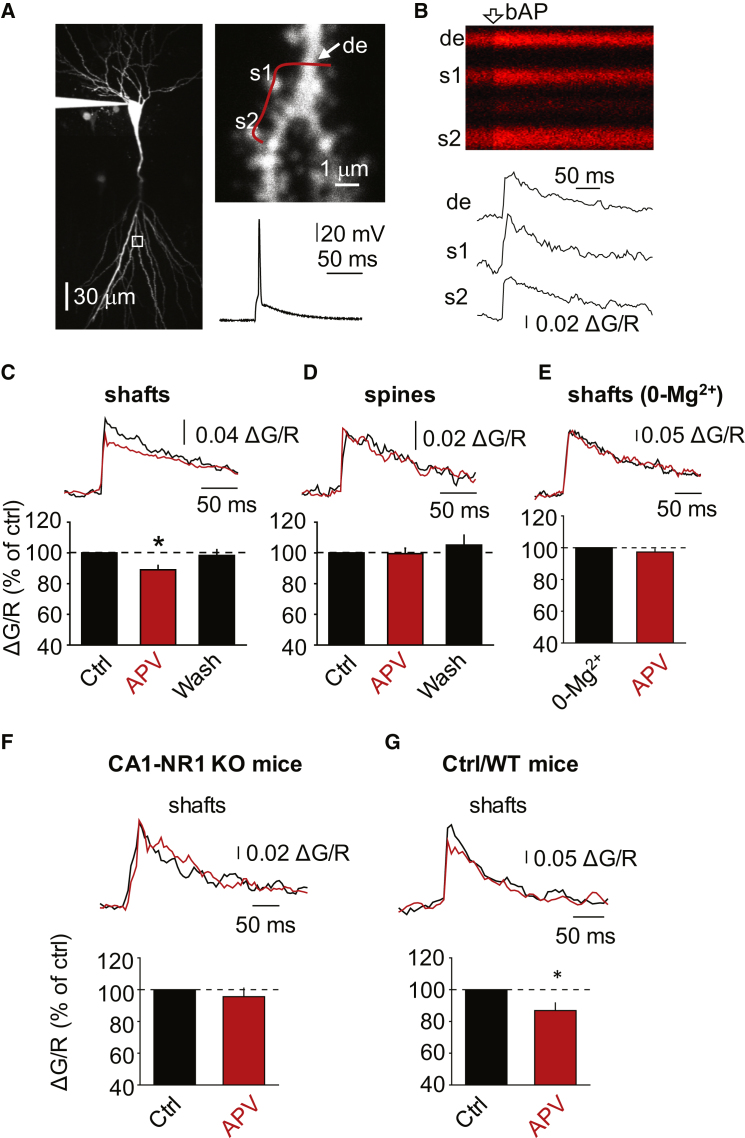
Activation of Shaft NMDARs Is Enabled by bAPs (A) Left panel shows a recorded neuron filled with Alexa Fluor 594. Upper right is the boxed region expanded. Red line indicates the line-scan trajectory through the dendritic shaft (de) and spines (s1, s2). Lower right illustrates somatic AP in response to current injection. (B) Line-scan Ca^2+^ imaging (upper) and average traces (lower); notation are as in (A). (C and D) The effect of NMDAR antagonist APV on bAP-evoked Ca^2+^ entry (*ΔG/R*) in shafts (C) and spines (D). Averaged traces in control (black) and in APV (red). (E) The effect of APV on bAP-evoked Ca^2+^ entry in Mg^2+^-free solution (0-Mg^2+^) in shafts. (F and G) The effect of APV on bAP-evoked Ca^2+^ entry (*ΔG/R*) in shafts of CA1-NR1 KO mice (F) and control (Ctrl) littermates (G). Summary data normalized to control (Ctrl or 0-Mg^2+^). Wash, washout of APV. Error bars in (C)–(G) indicate the SEM. ^∗^p < 0.05. See also Figure S1.

**Figure S1:**
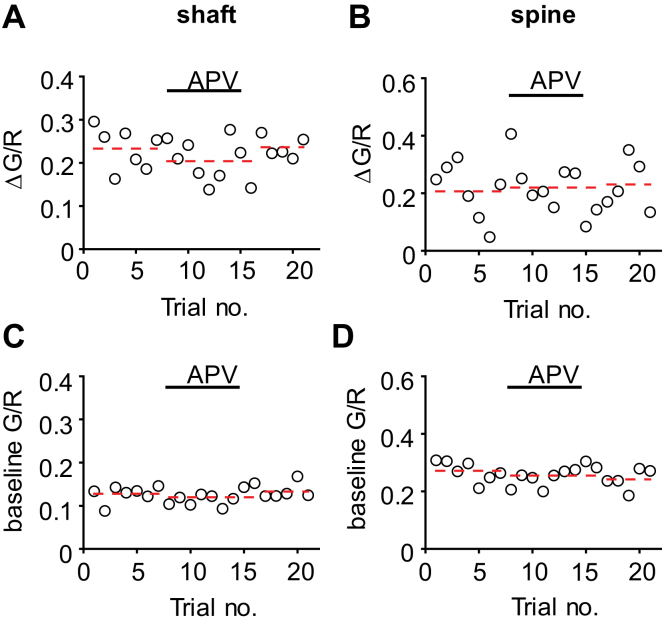
The Trial-by-Trial Time Course of the Effect of APV on bAP-Evoked Ca^2+^ Signals in Dendrites, Related to Figure 1 (A and B) APV slightly decreases bAP-evoked Ca^2+^ transients (*ΔG/R*) in the dendritic shaft (A) but not in spine (B). (C and D) Baseline Ca^2+^ indicated by *baseline G/R* is not affected by APV in the shafts (C) and spines (D). Open circles, individual trials. Red dash-lines indicate the mean values for each condition.

We held a hippocampal CA1 pyramidal cell in whole-cell current-clamp mode, filled it with the Ca^2+^ indicator Fluo-4 (250 μM) and the morphological tracer Alexa Fluor 594 (20–50 μM), and imaged Ca^2+^ transients induced by a single bAP in shafts and spines of the apical oblique dendrites (Figures 1A and 1B; AMPA, kainate, and GABA_A_ receptors were blocked). The amplitude of fluorescence Ca^2+^ responses (*ΔG/R*; Experimental Procedures) in the dendritic shafts was reversibly reduced to 89% ± 3% of baseline by the broad-spectrum NMDAR antagonist APV (50 μM) (n = 13, p = 0.001; Table 1; Figure 1C; Figure S1). Strikingly, we detected no such reduction in the spines on the same dendritic shaft (p = 0.31; Table 1; Figures 1D and S1). Because the overwhelming majority of excitatory synapses in CA1 pyramidal cells are hosted by spines (Bourne and Harris, 2011), this result suggests that extrasynaptic, rather than synaptic, NMDARs are activated upon generation of a bAP. The lack of APV effects on the bAP-evoked Ca^2+^ influx in spines also suggests that unblocking NMDARs had no detectable influence on the bAP waveform (e.g., amplitude or duration) that controls the opening of local VDCCs.

Activation of NMDARs could be enhanced by voltage-dependent channel gating upon depolarization directly, even in the absence of the Mg^2+^ block (Clarke and Johnson, 2008). However, removal of Mg^2+^ from the extracellular solution completely abolished the effect of APV on bAP-evoked Ca^2+^ entry (*ΔG/R*; 97% ± 2% of control, n = 6, p = 0.09; Figure 1E), arguing against any contribution of the voltage-dependent receptor properties other than the Mg^2+^ block. This experiment also rules out nonspecific network actions of the NMDAR blockade (e.g., suppression of spontaneous synaptic release).

We carried out another experiment to confirm that the aforementioned effects depend on the presence of functional dendritic NMDARs in CA1 pyramidal cells rather than on the network consequences of APV actions. The bAP-evoked Ca^2+^ entry in slices prepared from mice with a conditional deletion of the NR1 subunit of NMDAR restricted to CA1 pyramidal neurons (Tsien et al., 1996) was insensitive to APV application (*ΔG/R*; shafts: 96% ± 5% of control, n = 6, p = 0.45; Figure 1F). At the same time, APV was effective in the littermates that expressed functional NMDARs (*ΔG/R*; shafts: 87% ± 5% of control, n = 6, p = 0.04; Figure 1G).

**Figure 2:**
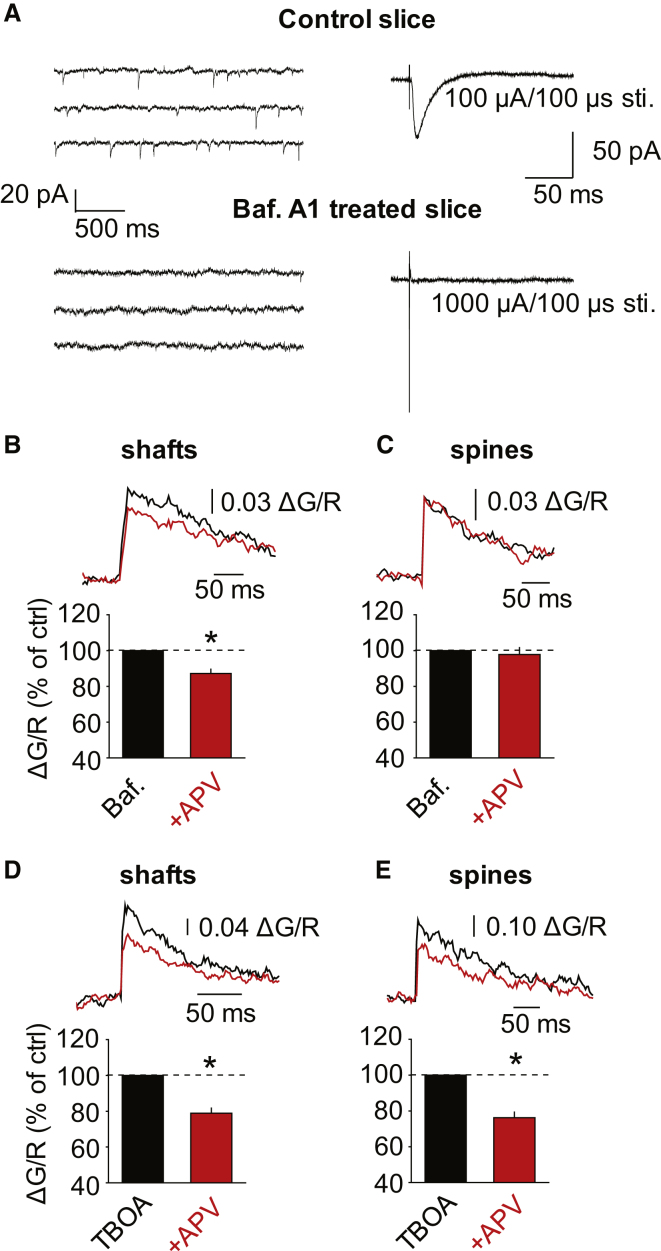
NMDAR Contribution to bAP-Evoked Ca^2+^ Transients in Dendritic Spines and Shafts under Blockade of Vesicular Release and Glutamate Uptake (A) Spontaneous and evoked synaptic activity blocked by bafilomycin A1. Upper panel shows traces with spontaneous EPSCs (left) and evoked EPSC (right) recorded in control slice. Lower panel illustrates traces without spontaneous and evoked EPSCs in bafilomycin A1-treated slice. sti., stimulus. (B and C) The effect of APV on bAP-evoked Ca^2+^ entry (*ΔG/R*) in shafts (B) and spines (C) of CA1 pyramidal neurons from bafilomycin A1-treated slices. Upper panels show averaged traces of bAP-evoked Ca^2+^ transients in control (black trace) and after adding APV (red trace) in one characteristic dendritic shaft and spine, respectively. Lower panels are summary data normalized to Baf. (i.e., control state of bafilomycin A1-treated slice). (D and E) The glutamate transporter blocker TBOA increases the effect of APV on bAP-evoked Ca^2+^ transients (*ΔG/R*) in shafts (D) and reveals an APV-sensitive component in Ca^2+^ transients in spines (E). Upper panels show averaged traces of bAP-evoked Ca^2+^ transients in TBOA (black trace) and in TBOA +APV (red trace) in one characteristic dendritic shaft and spine, respectively. Lower panels are summary data normalized to the Ca^2+^ transient in TBOA. Error bars in (B)–(E) indicate the SEM. ^∗^p < 0.05. See also Figure S2.

Next, we addressed the possibility that the APV sensitivity of bAP-evoked Ca^2+^ entry in dendritic shafts could be due to diffusion of Ca^2+^ from the spines where glutamate was released during bAP and that were not sampled. Indeed, bAP may coincide with presynaptic glutamate release and activate synaptic NMDARs, but contribution of such events to the shaft Ca^2+^ transients is highly unlikely. First, spines are considered to be relatively isolated Ca^2+^ compartments (Sabatini et al., 2002; Yuste and Denk, 1995). Second, in baseline conditions the frequency of spontaneous synaptic discharges detected using whole-cell recordings was 1–3 Hz (Figure 2A). Given the 5,000–30,000 excitatory synapses hosted by each CA1 pyramidal cell (Megías et al., 2001), spontaneous discharge occurs at each individual synapse once every hour or so. Indeed, we saw no spontaneous Ca^2+^ signals in 111 recorded postsynaptic spines each monitored over several minutes. Therefore, it is unlikely that baseline spontaneous synaptic activity can have detectable impact on shaft Ca^2+^ signals that we recorded. Finally, we tested the contribution of synaptic activity by blocking vesicular release of glutamate with 4 μM bafilomycin A1 (a specific inhibitor of vacuolar-type H^+^-ATPase; Experimental Procedures). Indeed, this treatment completely abolished both spontaneous synaptic events and evoked synaptic responses (Figure 2A). Strikingly, we found a qualitatively identical effect of APV on bAP-evoked Ca^2+^ entry in these slices compared to control conditions (Table 1; Figures 2B and 2C). These observations indicated that vesicular release of glutamate does not contribute significantly to the APV sensitivity of bAP-evoked Ca^2+^ entry in the shafts. This was also consistent with previous reports demonstrating that ambient glutamate in a quiescent slice has a nonsynaptic origin (Cavelier and Attwell, 2005; Fleming et al., 2011; Jabaudon et al., 1999; Le Meur et al., 2007).

### Glutamate Transporters Protect Postsynaptic NMDARs from Ambient Glutamate

**Figure S2:**
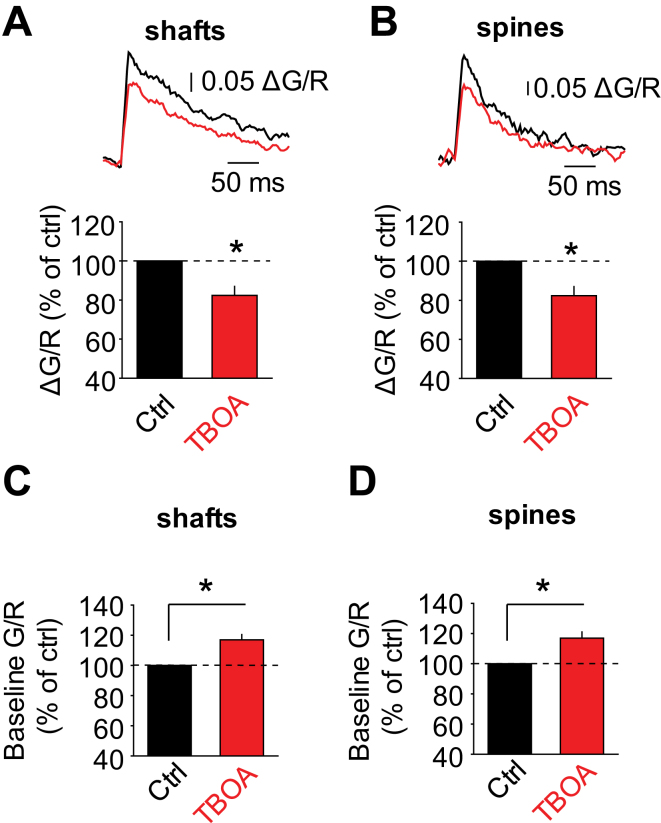
The Glutamate Uptake Blocker TBOA Reduces bAP-Evoked Ca^2+^ Transients, Related to Figure 2 (A and B) TBOA reduces bAP-evoked Ca^2+^ transients(*ΔG/R*) in dendritic shafts (A) (80 ± 3% of control; n = 9, p < 0.001) and spines (B) (80 ± 5% of control in spines; n = 18, p < 0.001). *Upper panels*, Ca^2+^ transients in control (black line) and in TBOA (red line) in shafts (A) and spines (B). *Lower panels*, summary data normalized to control (‘Ctrl’). (C and D) TBOA increases baseline Ca^2+^ (*baseline G/R*) both in dendritic shafts (C) and spines (D). (A–D) Error bars indicate the SEM. ^∗^, p < 0.05.

Electron microscopy suggests that astrocytic processes, which are enriched in high-affinity glutamate transporters (Lehre and Danbolt, 1998) and provide >90% of the glutamate uptake in area CA1 (Danbolt, 2001), tend to occur in the vicinity of postsynaptic spines (Lehre and Rusakov, 2002). Although this transporter shield provides a powerful buffer for glutamate that escapes from the adjacent synaptic cleft (Bergles et al., 1999; Diamond and Jahr, 1997), it could also protect local synaptic NMDARs from extracellular glutamate originating from outside the immediate synapse. We tested this hypothesis by blocking glutamate uptake and measuring NMDAR contribution into the bAP-evoked Ca^2+^ entry in dendritic shaft and associated spines. Indeed, in the presence of 100 μM TBOA, a potent glutamate transporter blocker, the NMDARs had similar contribution to the bAP-induced Ca^2+^ transients in both dendritic spines and shafts (Table 1; Figures 2D and 2E), consistent with the removal of transporter “protection” (Lozovaya et al., 2004a; Scimemi et al., 2004). We also detected that in the presence of TBOA, the APV-sensitive component of bAP-evoked Ca^2+^ entry in the dendritic shaft had increased compared to baseline conditions (Table 1; Figure 2D), probably due to an overall increase in extracellular glutamate. We also noticed that TBOA somewhat increased the baseline Ca^2+^ fluorescence, likely due to increased sustained activation of NMDARs (Figure S2). The consequent Ca^2+^ dye saturation could explain why the bAP-evoked Ca^2+^ fluorescent increments were somewhat decreased in TBOA, both in dendrites and spines. Although these data are consistent with the hypothesis that glutamate transporters protect synaptic NMDARs from exposure to glutamate, it was important to demonstrate this phenomenon without the potential concomitants of the TBOA action.

### Shaft and Spine NMDARs Can Detect Glutamate Escaping from Active Synapses

**Figure S3:**
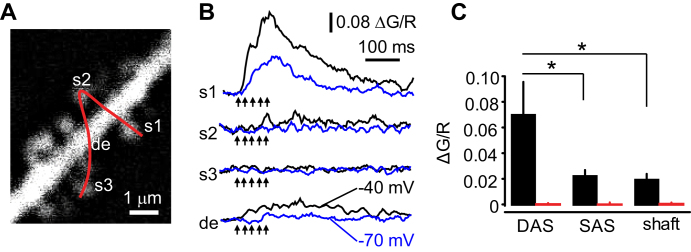
Ca^2+^ Response to Synaptically Released Glutamate in Dendritic Shafts and Spines, Related to Figure 3 (A) A recorded dendrite. Red line: line-scan position through the spines (*s1-3*) and the shaft (*de*). (B) Ca^2+^ transients (*ΔG/R*) in corresponding shaft and spines induced by 5 × 50 Hz electric stimulation (black arrows) when the cell was voltage-clamped at −70 mV (blue line) or at −40 mV (black line). Large responses were detected in *s1* (directly activated spine - *DAS*); a small response in *s2* (spillover activated spine - *SAS*) and *de*; no response in *s3* (inactive spine). (C) Averaged amplitude of Ca^2+^ transients (*ΔG/R*) at −40 mV from different cells in *DASs* (n = 4), *SASs* (n = 15) and shafts (n = 9) in control conditions (black bars) and in the presence of APV (red bars). Error bars indicate SEM. ^∗^, p < 0.05.

We therefore tested whether glutamate transporters still “protect” synaptic NMDARs under moderate network activity by applying a brief train of stimuli to Schaffer collaterals (five at 50 Hz; Experimental Procedures): this stimulation is compatible with physiological discharges of CA3 pyramidal cells and is thought to be sufficient to produce detectable glutamate escape (Lozovaya et al., 2004b; Scimemi et al., 2004). When the cell was held at −70mV with AMPA receptors (AMPARs) intact, this stimulus evoked clear Ca^2+^ responses in a proportion of dendritic spines, but not in dendritic shafts (Figure S3). This pattern of responses has routinely been associated with spines activated by glutamate released at the immediate synapse (Sabatini et al., 2002). Next, we depolarized the cell to −40mV to relieve the Mg^2+^ block of NMDARs. Under these conditions synaptic stimulation did evoke a detectable APV-sensitive Ca^2+^ response in a proportion of the previously unresponsive spines, as well as in the dendritic shaft (Figure S3). The most plausible explanation is that removing the Mg^2+^ block has boosted the response of spine and shaft NMDARs to glutamate escaping from active synapses. An alternative explanation involving activating NMDAR-only (“silent”) synapses (Kerchner and Nicoll, 2008) is unlikely because the “mature” spine types that we routinely image in adult animals are thought to host synapses equipped with AMPARs (Matsuzaki et al., 2001)

**Figure 3:**
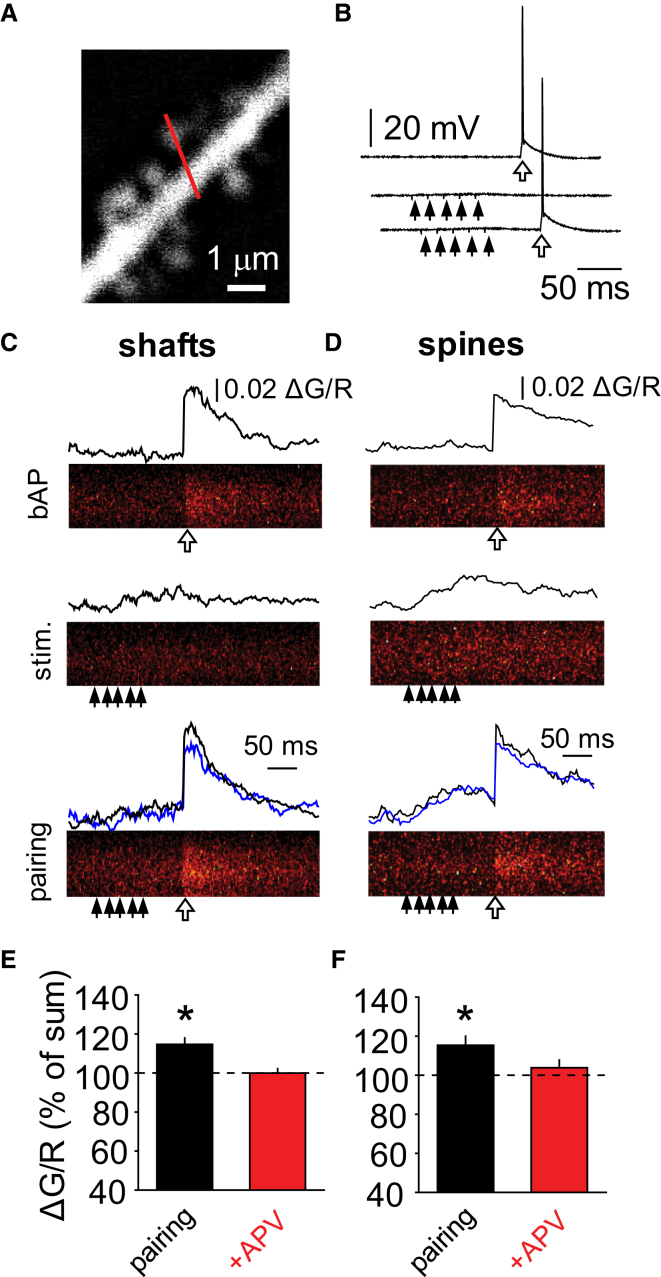
During Repetitive Synaptic Stimulation, bAPs Enable Detection of Glutamate Escape by Both Shaft and Spine NMDARs (A) A recorded dendrite with line-scan positions (red line). (B) Somatic response to current injection (open arrows), local synaptic stimulation (black arrows), and their combination. (C and D) Line-scan images and the corresponding traces (black) of Ca^2+^ transients in shafts (C) and spines (D) induced by a bAP (top), synaptic stimulation (stim.; middle), and synaptic stimulation paired with a bAP (pairing; bottom). Blue lines indicate arithmetic sum of bAP and stim. traces. (E and F) Summary data of pairing response normalized to the sum of bAP and stim. Responses in shafts (E) and spines (F) at baseline (pairing) and in APV (+APV). Error bars indicate the SEM. ^∗^p < 0.05. See also Figure S3.

To understand the role of signals mediated by synaptic glutamate escape, we therefore focused on the spines showing small (indirectly activated) NMDAR-mediated Ca^2+^ responses, termed here as spillover-activated spines (SASs). In the presence of AMPAR blocker NBQX, synaptic stimulation paired with a bAP (70 ms apart, Figures 3A and 3B) led to a relatively small, but highly significant, supralinear summation of Ca^2+^ entry both in the shafts (*ΔG/R*; 115% ± 3% of the sum, n = 10, p < 0.001; Figures 3C and 3E) and in the spines (*ΔG/R*; 115% ± 5% of the sum, n = 19, p = 0.001; Figures 3D and 3F; cells were held in current-clamp mode). If anything the supralinear effect is likely to be underestimated under these conditions because any partial saturation of the fluorescence indicator would produce a smaller fluorescence increment in response to the same Ca^2+^ entry. Importantly, the effect was completely abolished by APV (*ΔG/R*; shafts: 100% ± 2% of the sum, n = 7, p = 0.49; spines: 104% ± 4% of the sum, n = 13, p = 0.17; Figures 3E and 3F), suggesting that bAPs can provide a readout mechanism for detection of glutamate by both shaft and spine NMDARs.

### Shaft and Spine NMDARs Detect Extracellular Glutamate Released from a Volume-Limited Source

**Figure 4:**
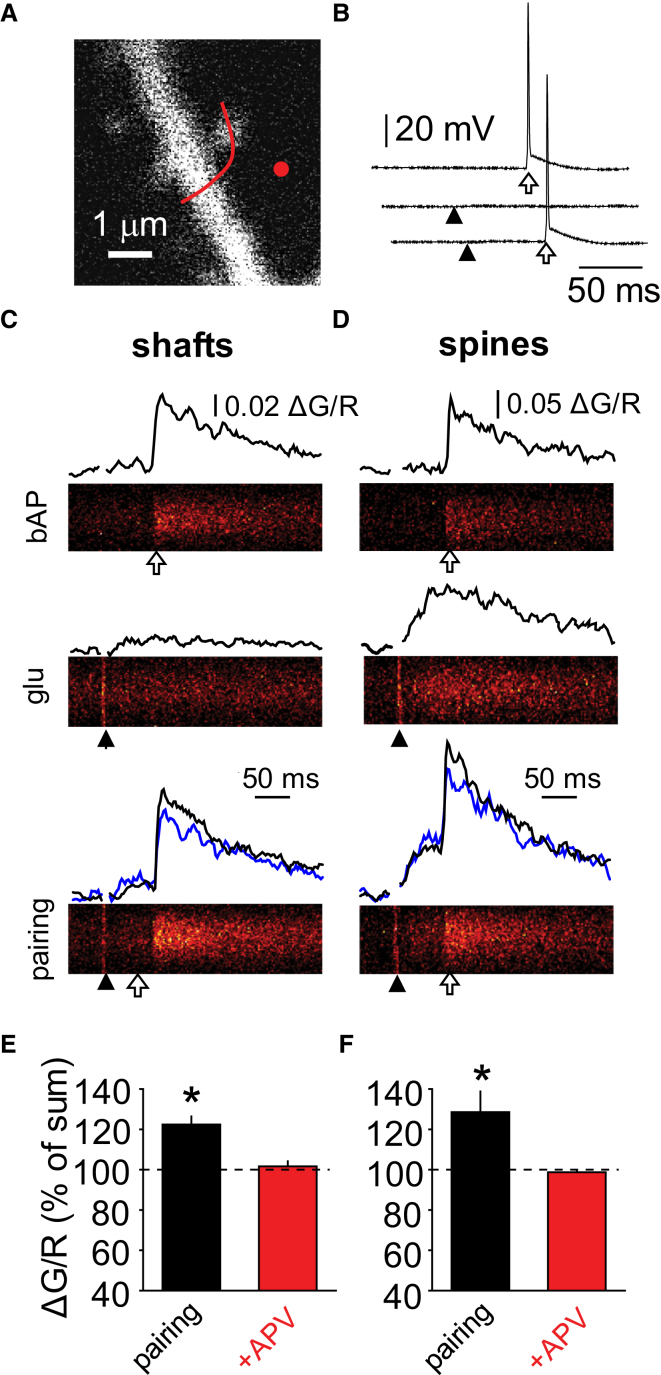
bAPs Trigger Detection of Photolysis-Induced Local Rises in Extracellular Glutamate by Both Shaft and Spine NMDARs (A) A recorded dendrite depicting line-scan positions (red lines). Red circle indicates the uncaging spot. (B) Somatic response to current injection (open arrows), local glutamate uncaging (black arrowheads), and their combination. (C and D) Line-scan images and the corresponding traces (black) of Ca^2+^ transients in shafts (C) and spines (D) induced by a bAP (top), uncaging (glu; middle), and uncaging paired with a bAP (pairing; bottom). Blue lines indicate arithmetic sum of bAP and glu traces. (E and F) Summary data of pairing response normalized to the sum of bAP and glu responses at baseline (pairing) and in APV (+APV). Error bars indicate the SEM. ^∗^p < 0.05.

Stimulation of afferent fibers activates multiple sources of glutamate in the neuropil in a relatively indiscriminate manner, making it impossible to gauge typical distances between the source and the detected Ca^2+^ signal. To control glutamate release in space and time, we employed two-photon uncaging of extracellular glutamate. To test the sensitivity of the bAP-evoked Ca^2+^ entry to local glutamate rises, we uncaged glutamate at a single point 1 μm away from both the spine and the parent shaft (5 ms pulse), a distance exceeding the average nearest-neighbor distance between synapses in the hippocampus (∼0.5 μm) (Rusakov and Kullmann, 1998) (Figures 4A and 4B). The uncaging of glutamate in the presence of an AMPAR antagonist produced small Ca^2+^ transients, which are likely to reflect the fraction of NMDARs Mg^2+^ unblocked at resting conditions (Kovalchuk et al., 2000); these transients were completely blocked by APV (Figure S3). When, however, uncaging was paired with a bAP, the resulting Ca^2+^ signals were again substantially higher than the sum of the Ca^2+^ signals evoked by either uncaging or a bAP alone (*ΔG/R*; shafts: 122% ± 4% of the sum, n = 7, p < 0.001; Figures 4C and 4E and spines: 129% ± 10% of the sum, n = 7, p = 0.015; Figures 4D and 4F). Again, the supralinearity was completely abolished by APV (*ΔG/R*; shafts: 102% ± 3% of the sum, n = 7, p = 0.28; Figure 4E and spines: 99% ± 1% of the sum, n = 7, p = 0.19; Figure 4F). This result is therefore consistent with our suggestion that bAPs can provide a readout of local extrasynaptic glutamate rises, be it from synaptic activity (Rusakov and Kullmann, 1998; Zheng et al., 2008), or through astrocytic (Jabaudon et al., 1999) or ectopic dendritic (Duguid et al., 2007; Shin et al., 2008) release.

### Burst Firing Enhances Ca^2+^ Entry through Extrasynaptic NMDARs

**Figure 5:**
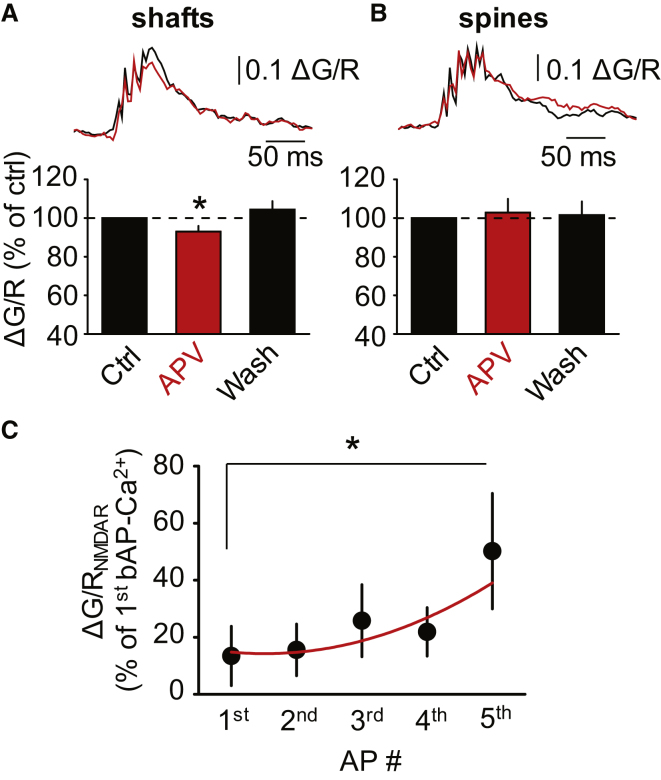
Burst Firing Sustains Sensitivity of Shaft NMDARs to Ambient Glutamate under Increased Ca^2+^ Entry (A and B) The effect of APV on burst bAP-evoked Ca^2+^ transients (*ΔG/R*) in shafts (A) and spines (B). Upper panels show averaged traces of burst bAP-evoked Ca^2+^ transients at baseline (black trace) and in APV (red trace) in one characteristic dendritic shaft and spine, respectively. Lower panels are summary data normalized to the burst bAP-evoked Ca^2+^ transient in control (Ctrl). (C) More NMDAR-mediated Ca^2+^ entry is triggered with increased number of bAPs within a burst. *ΔG/R_NMDAR_*, NMDAR-mediated Ca^2+^ entry; AP #, sequential number of APs within a burst. Error bars indicate the SEM. ^∗^p < 0.05. See also Figure S4.

**Figure S4:**
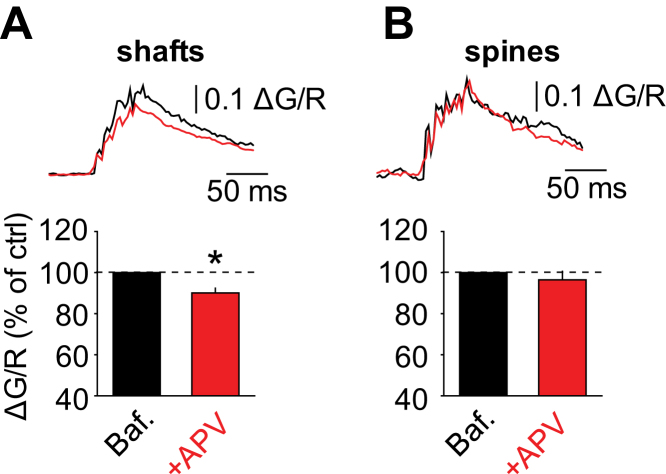
Burst Firing Triggers Detection of Ambient Glutamate by Shaft, but Not Spine NMDARs in Bafilomycin A1-Treated Slice, Related to Figure 5 (A and B) The effect of APV on burst bAPs-evoked Ca^2+^ entry (*ΔG/R*) in shafts (A) and spines (B) of CA1 pyramidal neurons from bafilomycin A1 treated slices. *Upper panels*, averaged traces of burst bAPs-evoked Ca^2+^ transients in control (black trace) and after adding APV (red trace) in one characteristic dendritic shaft and spine, respectively. *Lower panels*, summary data normalized to ‘Baf.’– control state in bafilomycin A1 treated slice. Error bars indicate SEM. ^∗^, p < 0.05.

What could be the physiological consequences of the dendritic Ca^2+^ entry enhanced by bAPs? Does a bAP recruit all glutamate-bound extrasynaptic NMDARs, or is there a room for signal modulation? If bAP recruits only a proportion of receptors, burst firing of the cell should produce larger Ca^2+^ entry through NMDARs due to the resulting larger dendritic depolarization. To address this, we monitored Ca^2+^ entry mediated by the burst of bAPs (using the low-affinity Ca^2+^ dye Fluo-4FF to approach the linear sensitivity range during burst stimulation). We found that the relative effects of APV on Ca^2+^ entry were similar for 5 × 100 Hz bursts and single bAPs (*ΔG/R*; shafts: 93% ± 3% of control, n = 10, p = 0.02; and spines: 103% ± 7% of control, n = 10, p = 0.35; Figures 5A and 5B). However, because of increased depolarization, burst firing can also enhance the contribution of VDCCs to Ca^2+^ entry. Therefore, we measured APV-sensitive response on each stimulus in the burst (NMDAR mediated bAP-Ca^2+^; *ΔG/R_NMDAR_*) and normalized it to the amplitude of total Ca^2+^ response to the first bAP. This measurement indicated that a larger number of NMDARs are indeed recruited with more bAPs in a burst (*ΔG/R_NMDAR_*: 5^th^ versus 1^st^ bAP, n = 10, p = 0.04; Figure 5C). Strikingly, a similar result was obtained in slices pretreated with bafilomycin A1 (*ΔG/R*; shafts: 90% ± 2% of control, n = 8, p = 0.002; spines: 97% ± 4% of control, n = 7, p = 0.21; Figure S4). Thus, synaptically released glutamate does not contribute to activation of extrasynaptic NMDARs in the slice even during the burst firing of the postsynaptic cell.

### Repeated Burst Spiking Downregulates *G*_h_ by Engaging Extrasynaptic NMDARs

**Figure 6:**
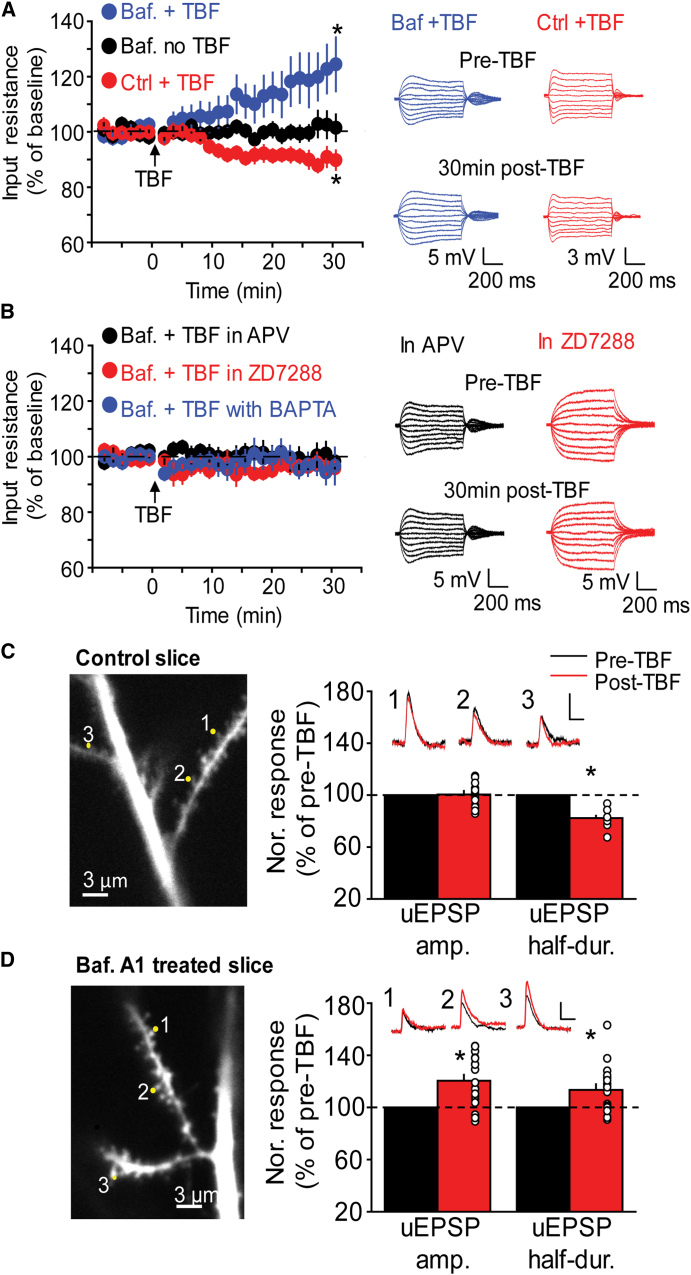
Activation of Extrasynaptic NMDARs during TBF Downregulates *G*_h_ and Enhances uEPSPs (A) Left panel illustrates the changes in cell input resistance induced by TBF in bafilomycin A1-treated slice (blue circles) and control slice (red circles). No gradual change in input resistance was detected in bafilomycin A1-treated slice without TBF (black circles). Right panel shows voltage response to current injections before (pre-TBF) and 30 min after (post-TBF) TBF in bafilomycin A1-treated (blue traces) and control (red traces) slices. (B) Left panel shows that TBF did not produce detectable change in the input resistance in bafilomycin A1-treated slice in the presence of APV (black circles) or ZD7288 (red circles) or dialyzing the cell with BAPTA (blue circles). Right panel illustrates voltage response to current injections before (pre-TBF) and 30 min after (post-TBF) TBF in the presence of APV (black traces) and ZD7288 (red traces). (C) Left panel illustrates that glutamate was uncaged on spines of apical dendrites of CA1 pyramidal neuron in control slices. Right panel is the summarized results of the amplitudes and half-durations of uEPSP before (black) and after (red) TBF. Insets show the uEPSP traces recorded via a somatic whole-cell patch pipette before (black) and 30 min after (red) the induction of TBF. Calibration, 50 ms, 1mV. (D) The experiment settings were similar to those in (C) but performed in bafilomycin A1-treated slices. Error bars indicate the SEM. ^∗^p < 0.05. See also Figure S5.

**Figure S5:**
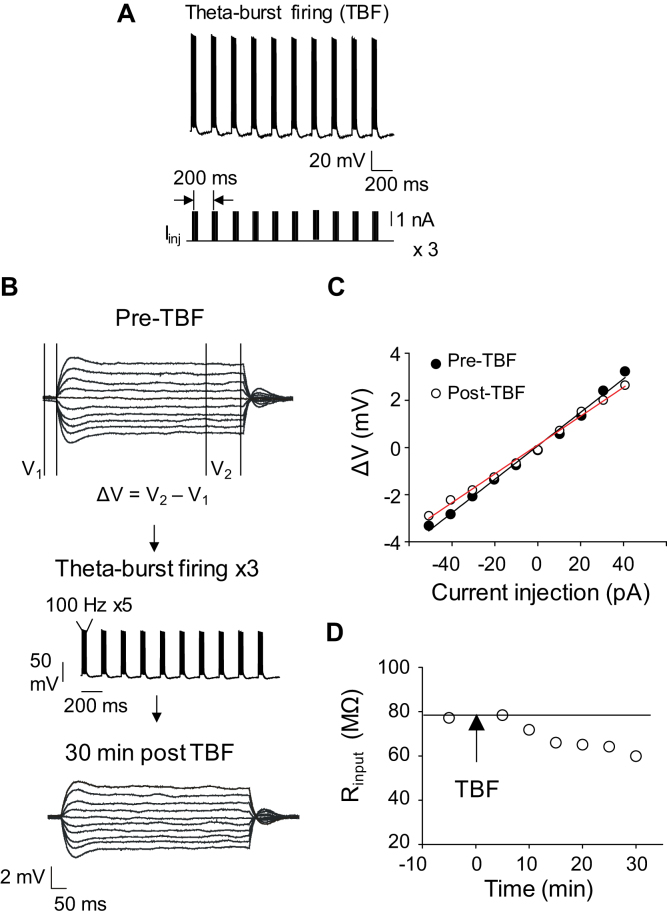
The Protocol of Theta-Burst Firing Stimulation and Measurement of Cell Input Resistance by Steps of Current Injection, Related to Figure 6 (A) Protocol of theta-burst firing (TBF) stimulation. *Upper trace*, an example of somatic membrane potential during TBF stimulation. *Lower trace*, the amplitude of the injected current during TBF. (B) A demonstration of the measurement of cell input resistance (R_input_) and the protocol to induce R_input_ plasticity. ΔV is the membrane potential changes upon 700 ms current injection. (C) R_input_ is determined by fitting the linear I-V relation (current injection v.s. ΔV) upon current injections. Here shows an example of the fitting pre- (black line) and post-TBF (red line). (D) An example of a single experiment of R_input_ plasticity after TBF.

In many cases postsynaptic Ca^2+^ elevation triggers various forms of cellular plasticity. We therefore asked whether activation of glutamate-bound extrasynaptic NMDARs during burst activity has any lasting consequences for neuronal excitability. It has previously been shown that activation of glutamate-bound synaptic NMDARs during theta-burst firing (TBF) (Figure S5) can reduce input resistance and excitability of CA1 pyramidal neurons because of *G*_h_ upregulation (Fan et al., 2005). We repeated these experiments under similar control conditions and in slices pretreated with bafilomycin A1. In control slices, TBF led to a gradual decrease in the cell input resistance (to 90% ± 4% of baseline 30 min post-TBF, n = 8; p = 0.02; Figure 6A), consistent with the previously reported upregulation of *G*_h_ (Fan et al., 2005). In striking contrast, similar stimulation in slices treated with bafilomycin A1 increased input resistance (to 124% ± 9% of baseline 30 min post-TBF, n = 6; p = 0.046), whereas bafilomycin A1 alone had no effect (Figure 6A). The effect of TBF on bafilomycin A1-treated slices was completely abolished either by the NMDAR antagonist APV (98% ± 3% of baseline in 30 min after TBF, n = 5; p = 0.52; Figure 6B), by the *h*-channel blocker ZD7288 (20 μM, 96% ± 2% of baseline in 30 min after TBF, n = 5; p = 0.14; Figure 6B), or by chelating intracellular Ca^2+^ with 10 mM BAPTA (97% ± 7% of baseline in 30 min after TBF, n = 7, p = 0.37; Figure 6B). These experiments suggest that Ca^2+^ entry during activation of extrasynaptic NMDARs by bursts of bAP is responsible for downregulation of *G*_h_.

Changes in *G*_h_ and associated changes in input resistance can affect the synaptic input into cell dendrites (Campanac et al., 2008; Fan et al., 2005). Local spot uncaging near identified dendritic spines produced EPSP-like potential (uEPSP) in the cell soma (Figures 6C and 6D). Consistent with previous reports, upregulation of *G*_h_ in control slices did not significantly affect the amplitude of the uEPSP (amplitude after TBF was 101% ± 3% of control, n = 12, p = 0.87, paired t test); however it significantly reduced the half-duration of the uEPSP (half-duration after TBF was 82% ± 2% of control, n = 12, p < 0.001, paired t test) (Figure 6C) (Magee, 1998; Poolos et al., 2002). TBF in bafilomycin A1-treated slices increased both the amplitude (amplitude after TBF was 121% ± 4% of control, n = 18, p < 0.001, paired t test) and the half-duration of the uEPSP (half-duration after TBF was 113% ± 4% of control, n = 18, p < 0.001, paired t test) (Figure 6D). This finding demonstrates a form of neuronal nonsynaptic plasticity induced by the readout of extrasynaptic glutamate by bAPs, which in turn affects integration of synaptic inputs in the postsynaptic cell.

## Discussion

### Shaft and Spine NMDARs Detect Ambient Glutamate Differently

We have found that a proportion of NMDARs located in dendritic shafts, but not spines, are bound to glutamate under resting conditions, enabling receptor activation by bAPs. Because glutamatergic synapses on CA1 pyramidal neurons occur mainly on dendritic spines, shaft NMDARs represent overwhelmingly extrasynaptic receptors (Petralia et al., 2010). Dendritic spines, however, may host both synaptic and extrasynaptic NMDARs. Because it may be difficult to distinguish between the two, we have focused on the physiological role of shaft NMDARs that are almost exclusively extrasynaptic. One plausible mechanism behind the functional distinction between spine and shaft NMDARs is the differential expression of local high-affinity neuronal and glial glutamate transporters. The relatively tight glial coverage of dendritic spines and the strong presence of highly efficient postsynaptic transporters appear to maintain a negligible background glutamate concentration inside the synaptic cleft in the absence of synaptic events (Diamond, 2001). Indeed, the blockade of glutamate uptake with TBOA revealed contribution of spine NMDARs in Ca^2+^ entry induced by bAPs. The relative isolation of the synaptic cleft from baseline ambient glutamate may help to distinguish between signals mediated by synaptic and extrasynaptic NMDARs. Furthermore, this isolation could help to minimize the desensitization of synaptic AMPARs by ambient glutamate (Trussell and Fischbach, 1989).

### Synaptic and Nonsynaptic Sources of Extracellular Glutamate

We found that the average NMDAR occupancy by glutamate in a quiescent acute slice does not depend on vesicular release, which is fully consistent with previous reports (Cavelier and Attwell, 2005; Jabaudon et al., 1999; Le Meur et al., 2007). The resulting space-and-time average extracellular glutamate concentration can be thought of as a “floor” level of ambient glutamate that is independent of the synaptic network activity. In addition to ambient nonvesicular glutamate, extrasynaptic NMDARs can also be bound to glutamate escaping from the synaptic cleft when synaptic network activity increases (Chalifoux and Carter, 2011; Scimemi et al., 2004). We found that synaptic discharges (or local glutamate uncaging mimicking such) paired with bAPs boost Ca^2+^ entry in both shafts and SASs (Figures 3 and 4). Thus, both shaft and spine NMDARs can also sense extracellular glutamate that is transiently elevated as a result of local synaptic activity but require a readout signal, such as the bAP, to be activated. Importantly, the coincidence detection interval for glutamate release and bAPs extends beyond the duration of an individual rapid glutamate rise, reflecting the fact that glutamate molecules can remain bound to dendritic NMDARs for hundreds of milliseconds. The NMDAR-mediated enhancement of bAP-evoked dendritic Ca^2+^ signals could therefore act as an integrating detector of glutamate release events that occurred nearby over an extended period of time.

### Downregulation of *G*_h_ Depends on Nonvesicular Release of Glutamate

The amount of Ca^2+^ entering the cell through bAP-enabled NMDARs is determined by the number of glutamate-bound NMDARs as well as by the proportion of these receptors unblocked by a bAP. Because the kinetics of the voltage-dependent NMDAR Mg^2+^ “unblock” are complex and include slow components (Kampa et al., 2004), broader bAPs are likely to recruit more NMDARs. Consequently, the bAP broadening during spike bursts could boost NMDAR-dependent Ca^2+^ entry in a supralinear fashion.

We have found that recruitment of shaft NMDARs by theta bursts of bAPs downregulates *G*_h_. In contrast, the recruitment of synaptic NMDARs by bAPs upregulates *G*_h_. We observed that dendritic input mimicked by local glutamate uncaging inversely correlated with such changes in *G*_h_. When *G*_h_ was downregulated following theta-burst stimulation, EPSP-like responses produced by glutamate uncaging were increased. When *G*_h_ was upregulated, the responses were decreased. Thus, the net effect of bAPs on the cell's membrane resistance, and consequently on the mode of synaptic input integration, depends on the balance between glutamate-bound synaptic versus extrasynaptic NMDARs. In this way, increased synaptic network activity can tip the balance in favor of synaptic NMDARs, whereas decreased synaptic activity shifts it back to the extrasynaptic NMDARs. Similar to synaptic potentiation and depression, this bidirectional plasticity mechanism prevents the cell from progressive runaway excitation, thus providing a theoretically plausible basis for information coding in the network.

Recent reports suggest that dendritic branches, rather than individual synapses, are the primary functional units for long-term memory storage (Govindarajan et al., 2011; Losonczy et al., 2008; Makara et al., 2009). These studies used synaptic stimulation to demonstrate that dendritic branches operate as single computational units. The present results suggest therefore that extrasynaptic glutamate signaling acting via the dendritic shaft NMDARs could play a potentially important part in such integration. This raises a number of questions. For example, could synaptic input to the basal dendrites, strong enough to drive AP firing, cause an increase in *G*_h_ in the basal dendrites while decreasing *G*_h_ in the apical dendrites? Or would the synaptically driven increase in *G*_h_ be global enough to extend into the apical dendrites? Clearly, a dedicated systematic study is required to address these questions.

### Possible Roles of Extrasynaptic Glutamate Signaling in Neuronal Synchronization

The activation of extrasynaptic NMDARs by the astrocytic release of glutamate has been suggested to act as a mechanism for neuronal synchronization (Angulo et al., 2004; Fellin et al., 2004), and a recent discovery of the use-dependent release of the NMDAR coagonist D-serine from astrocytes provides a potential regulating mechanism for this “diffuse” form of signaling (Henneberger et al., 2010). Interestingly, it has been suggested that astrocytes can release glutamate both in a vesicular (Bezzi et al., 2004) and a nonvesicular manner (Cavelier and Attwell, 2005; Szatkowski et al., 1990). In addition to the astrocytes, other sources can potentially contribute to the local spatially distributed transient elevations in ambient glutamate (Semyanov, 2008). Ectopic dendritic release has been proposed as one of such sources (Duguid et al., 2007; Shin et al., 2008).The present study suggests that such (slow) extracellular glutamate signals, by acting predominantly on dendritic shaft NMDARs, may trigger downregulation of *G*_h_ in a group of neurons in a synchronized fashion. The latter could in principle provide a mechanism for metaplasticity changes that help to handle information in the network.

## Experimental Procedures

### Animals and Hippocampal Slice Preparation

Transverse hippocampal slices were prepared from 21- to 35-day-old Sprague-Dawley rats or 42- to 49-day-old CA1-NR1 KO mice (NR1 *fl/fl; CaMKII-Cre*) or littermate controls (NR1 *fl/fl*) (Tsien et al., 1996) in accordance with the RIKEN regulations. Animals were anesthetized with 2-Bromo-2-chloro-1,1,1-trifluroethane and decapitated. The brain was exposed, chilled with ice-cold solution containing 75 mM Sucrose, 87 mM NaCl, 2.5 mM KCl, 0.5 mM CaCl_2_, 1.25 mM NaH_2_PO_4_, 7 mM MgCl_2_, 25 mM NaHCO_3_, 1 mM Na-Ascorbate, and 11 mM D-glucose. Hippocampi from both hemispheres were isolated and placed in an agar block. Transverse slices (350–400 μm) were cut with a vibrating microtome (Microm HM 650V; Thermo Fisher Scientific) and left to recover for 30 min at 34°C and then at room temperature for 1 hr in interface chamber with “storage” solution containing 127 mM NaCl, 2.5 mM KCl, 1.25 mM NaH_2_PO_4_, 1 mM MgCl_2_, 1 mM CaCl_2_, 25 mM NaHCO_3_, and 25 mM D-glucose. Then the slices were transferred to the recording chamber and were continuously perfused at 34°C with a solution containing 127 mM NaCl, 2.5 mM KCl, 1.25 mM NaH_2_PO_4_, 1 mM MgCl_2_, 2 mM CaCl_2_, 25 mM NaHCO_3_, and 25 mM D-glucose. All solutions were saturated with 95% O_2_ and 5% CO_2_. Osmolarity was adjusted to 298 ± 3 mOsm. A total of 25 μM NBQX, 100 μM picrotoxin, 5 μM CGP52432, and 100 μM LY341495 (or 200 μM S-MCPG) was routinely added to the solution to block AMPA/kainate, GABA_A_, GABA_B_, and metabotropic glutamate receptors, respectively, unless stated otherwise.

To block the vesicular release of neurotransmitters, freshly prepared slices were incubated at 34°C for 2.5 hr in “storage” solution containing 4 μM of bafilomycin A1. The control slices for this set of experiments were incubated in the same conditions but without bafilomycin A1.

### Visualized Patch-Clamp Recording

CA1 pyramidal neurons were visually identified under infrared illumination using Olympus BX-61 microscope equipped with differential interference contrast optics and a water-immersion objective lens (60×, NA = 0.9; Olympus, Japan). The cells were approached with a patch pipette using motorized manipulators (Luigs & Neumann, Germany). For imaging experiments, whole-cell current-clamp recordings were obtained with a patch pipette (3–6 MΩ) filled with a solution containing 130 mM KCH_3_SO_3_, 8 mM NaCl, 10 mM HEPES, 10 mM Na_2_-Phosphocreatine, 0.4 mM Na_2_GTP, 4 mM MgATP, 3 mM Na-Ascorbate (pH 7.2), and osmolarity was adjusted to 290 mOsm. The recording solution also contained the morphological tracer Alexa Fluor 594 (20–50 μM, R channel) and the Ca^2+^-sensitive dye Fluo-4 (250 μM, G channel) or Fluo-4FF (500 μM, G channel).

bAPs were induced by somatic current injections (2–3 ms, 600–1,000 pA) and recorded in the soma with the patch amplifier (Multiclamp 700B; Axon Instruments, Union City, CA, USA). For plasticity experiments, input resistance was monitored in current-clamp mode, cells were recorded with pipette solution containing 130 mM K gluconate, 8 mM NaCl, 10 mM HEPES, 10 mM Na_2_-Phosphocreatine, 0.5 mM EGTA, 0.4 mM Na_2_GTP, 4 mM MgATP, 3 mM Na-Ascorbate (pH 7.2), and osmolarity was adjusted to 290 mOsm. Input resistance was determined from 700 ms current injections (ranging from −50 to +50 pA in steps of 10 pA every 3 s). The slop of linear fit of voltage changes versus the injected currents gave the value of the cell input resistance (Figure S5). TBF stimulation consisted of 30 trains of 5 APs firing at 100 Hz (10 trains at 5 Hz repeated 3 times with a 10 s interval) (Figure S5).

In voltage-clamp recordings the series resistance of the cells was measured by injection of hyperpolarizing pulses (−5mV, 100 ms) and was not compensated. The series resistances were usually <20 MΩ, and data were discarded if its value changed by more than 20% during the recording. In current-clamp recordings the series resistance was compensated with “bridge balance” function. Signals were filtered at 2 kHz and digitized at 4–10 kHz with NI PCI-6221 card (National Instruments). The data were recorded with software WinWCP and WinEDR (supplied free of charge to academic users by Dr. John Dempster, University of Strathclyde, UK).

### Two-Photon Imaging

Cells were filled with the dyes for at least 20 min before the start of recording. Two-photon Ca^2+^ imaging was performed with a two-scanner FV1000-MPE laser-scanning microscope (Olympus) equipped with a mode-locked (<140 fs pulse width) tunable 720–930 nm laser Chameleon XR (Coherent, USA). Both dyes were excited at 810 nm light wavelength, and their fluorescence was chromatically separated and detected with two independent photomultipliers (PMTs). We used the bright Alexa Fluor 594 emission to identify oblique apical dendrites (about 150 μm from the soma) and their spines. Line-scan imaging was performed to record Ca^2+^ signals in the dendritic shaft and one to four spines. Imaging was synchronized with electrophysiological recordings. At the end of each recording, we tested that Ca^2+^ transients were below Fluo-4 saturation level, which was achieved by prolonged somatic depolarization causing firing and Ca^2+^ buildup in the neurons. The changes in baseline Ca^2+^ level were monitored as ratio between baseline Fluo-4 and Alexa Fluor 594 fluorescences. If this ratio increased during the experiment for more than 20%, the cells were discarded. The dark noise of the PMTs was collected when the laser shutter was closed in every recording.

### Local Synaptic Stimulation and Finding the Active Spine

Local synaptic stimulation was done with an extracellular glass pipette placed 5–20 μm away from the apical oblique dendrite. The pipette had a tip diameter 2–3 μm and was filled with 1 M NaCl and 5 μM Alexa 594 to identify the pipette position. The spines were identified by Ca^2+^ transients in response to a train of five stimuli at 50 Hz in the absence of NBQX. Then experiments were performed in the presence of NBQX in the current-clamped cells. Three types of measurements were done in the dendritic shafts and the spines: (1) Ca^2+^ transients in response to a bAP; (2) Ca^2+^ response to synaptic stimulation; and (3) a response to the bAP and “synaptic” stimulation combined (“pairing”). In protocol “(3),” bAPs were initiated 70 ms after the end of synaptic stimulation.

### Glutamate Uncaging

4-methoxy-7-nitroindolinyl-caged L-glutamate (10 mM, MNI-glutamate) was applied locally via an extracellular glass pipette. The uncaging spot was located opposite to an oblique dendrite at equal distances from the imaged dendritic shaft and spine. Two-photon uncaging was carried out using mode-locked tunable 690–1,020 nm laser Mai-Tai (Spectra-Physics, USA). The uncaging was done in a “point scan” mode for 5 ms at 720 nm with FV1000-MPE system. The laser power was adjusted with acousto-optic modulator (AOM) to produce a just-detectable Ca^2+^ response (typically 2–3 mW). This illumination of the preparation in absence of MNI-caged glutamate, or the application of MNI-glutamate alone, had no effect on either the resting Ca^2+^ or Ca^2+^ transients induced by bAPs. For the “pairing” protocol the three types of recordings were carried out: (1) Ca^2+^ transients in response to a bAP; (2) a Ca^2+^ response to glutamate uncaging; and (3) a response to the bAP and uncaging combined. In protocol “(3),” bAPs were induced 70 ms after the uncaging pulse. In experiments addressing the physiological effects of TBF-induced plasticity, somatic uEPSPs were obtained by uncaging bath-applied MNI-glutamate (400 μM) using 5–10 ms laser pulses (405 nm diode laser; FV5-LD405; Olympus) at spots located close to spines on apical dendrites between 100 and 150 μm from the soma.

### Drugs and Chemicals

All drugs were made from stock solutions kept frozen at −20°C in 100–200 ml 1,000× aliquots. Picrotoxin, LY341495, S-MCPG, D-APV, NBQX, DL-TBOA, ZD7288, CGP52432, and MNI-caged glutamate were purchased from Tocris Cookson (Bristol, UK). Bafilomycin A1 was obtained from Wako Chemicals (Osaka, Japan). Chemicals for solutions were from Sigma-Aldrich (St. Louis). Alexa Fluor 594, Fluo-4, and Fluo-4FF were obtained from Invitrogen (Carlsbad, CA, USA).

### Data Analysis

Electrophysiological data were analyzed with WinWCP and Clampfit (Axon Instruments). Imaging data were analyzed using FluoView (Olympus), ImageJ (a public domain Java image processing program by Wayne Rasband), and custom software written in LabView (National Instruments, Austin, TX, USA). Statistical analysis was performed using Excel (Microsoft, USA) and Origin 8 (OriginLab).

The fluorescent measurements of Ca^2+^ transient were represented as *ΔG/R*: ((*G_peak_* − *G_baseline_*)/(*R_baseline_* − *R_dark noise_*)). Baseline Ca^2+^ signals were represented by *baseline G/R*, ((*G_baseline_* − *G_dark noise_*)/(*R_baseline_* − *R_dark noise_*)), where *G* is the Fluo-4 or Fluo-4FF fluorescence, and *R* is Alexa Fluor 594 fluorescence. *G_baseline_* and *R_baseline_* are averaged fluorescences 50–100 ms before the stimulation. *G_peak_* is averaged fluorescences 30–40 ms after the stimulation. *G_dark noise_* and *R_dark noise_* are the dark currents of the corresponding PMTs. For illustration purposes, single traces were processed by five-point moving average, and then four to five sequential traces were averaged.

The statistical significance was tested using a paired or unpaired Student's t test. The data are given in mean ± SEM. “n” designates the number of recordings. In all figures, error bars indicate mean ± SEM.

## Figures and Tables

**Table 1:** Effect of 50 μM APV on bAP-Evoked Ca^2+^ Transients

	Control	Bafilomycin A1	p Value	TBOA	p Value
Shafts	89 ± 3 (13)	87 ± 4 (4)	0.36	79 ± 3 (9)	0.011
Spines	98 ± 4 (22)	93 ± 5 (7)	0.22	77 ± 3 (18)	<0.001

Data presented as mean ± SEM (n, number); p values are given for difference with APV effect in control slices.

## Supplemental Information

Document S1. Article plus Supplemental Information
